# Maternal exposure to farming environment protects offspring against allergic diseases by modulating the neonatal TLR-Tregs-Th axis

**DOI:** 10.1186/s13601-018-0220-0

**Published:** 2018-08-17

**Authors:** Jinyan Yu, Xiaoqiu Liu, Yanlei Li, Shanshan Meng, Fei Wu, Bingdi Yan, Yanjun Xue, Tiangang Ma, Junling Yang, Jing Liu

**Affiliations:** 1 Department of Respiratory and Critical Care Medicine, The Second Hospital of Jilin University, 218 Ziqiang Street, Changchun, 130041 Jilin People’s Republic of China; 2 Department of Clinical Laboratory, The Second Hospital of Jilin University, 218 Ziqiang Street, Changchun, 130041 People’s Republic of China; 3 Department of Obstetrics and Gynecology, The Second Hospital of Jilin University, 218 Ziqiang Street, Changchun, 130041 People’s Republic of China; 4 Department of Critical Care Medicine, Zhongda Hospital, School of Medicine, Southeast University, Nanjing, People’s Republic of China

**Keywords:** Allergic diseases, Maternal farming exposure, Newborn, Tregs, TLR

## Abstract

**Background:**

As the development of urbanization in China, the morbidity of allergic disease rise up prominently even in children, which may be partially associated with the excessively clean environment. It has been reported that common microorganism in rural environment shows protective effects on allergic disease by modulating TLRs-Tregs/Th cell axis. But the mechanism of this protection still needs to be elucidated in detail. We investigated the effects of maternal exposure to farming environment on the neonatal innate immune system, especially on the TLR-Treg-Th (Th1, Th2, Th9, and Th17) axis, in the Jilin province of China.

**Methods:**

Eighty-four non-farming and 42 farming pregnant women were recruited. Endotoxins and glucans in dust from the living rooms of the pregnant mothers were measured. Cord blood mononuclear cells were challenged with phytohemagglutinin, lipopolysaccharide, or peptidoglycan. Proliferative response of lymphocyte was measured by 3H-TdR incorporation methods, CD4 + CD25 + FOXP3 + T cells percentage was assessed with flow cytometry, Tregs specific genes (FOXP3, LAG3, GITR, CTLA-4 and TGF-β) and TLR2, TLR4 genes expression were detected by RT-PCR, specific cytokines of Th1, Th2, Th9, Th17 and Tregs were measured with flow cytometer, suppressive capacity of Tregs was tested by culturing with effector cells in vitro, and TLR2/4 gene polymorphism was detected.

**Results:**

Higher endotoxin content was observed in the living rooms of the farming mothers. Compared with that in the non-farming group, in farming neonatal CBMCs, lymphocyte proliferation declined; the IFN-γ/IL-13 ratio increased; and the quantity of Tregs and gene expression of FOXP3, GITR, CTLA4 and TLR2 increased significantly (P < 0.05). Isolated Tregs suppressed the proliferation of effector T cells and IL-13 production more strongly in vitro (P = 0.04, 0.03, respectively), and the TLR2 polymorphism affected FOXP3 expression and IFN-γ and IL-13 production.

**Conclusions:**

Maternal exposure to farming affected the quantity and function of neonatal Tregs upon stimulation with PPG and LPS, which partly contributed to reducing the risk for allergic diseases in the offspring. The results of our study will lay the theoretical foundation for allergic disease prevention in early life.

**Electronic supplementary material:**

The online version of this article (10.1186/s13601-018-0220-0) contains supplementary material, which is available to authorized users.

## Background

With increasing urbanization, the incidence of allergic diseases such as asthma has increased worldwide, which has in turn sharply affected quality of life [1, 2]. China, in particular, is confronted with this severe problem, especially in some major cities such as Shanghai [3]. In early life, long-time over-loading of microorganisms in the environment, which includes contacting with livestock, or drinking fresh milk that containing multiple bacteria, endotoxins and fungi, has an important role in preventing allergic diseases such as childhood asthma [4, 5]. Exposure to microorganisms during infancy, or even at the fetal stage (via maternal exposure), decreases the morbidity due to allergic diseases in children [6–9]. However, research on the effects of maternal exposure to microbes in rural areas on the neonatal immune system and allergic diseases in infants is still lacking in China.

Toll-like receptors (TLRs) of the immune system are involved in recognizing microorganisms; their expression and gene polymorphisms are involved in the process of neonatal immune maturation [10]. The major ligands of TLR2 and TLR4 are peptidoglycans (PPG) and lipopolysaccharide (LPS), respectively. TLR2 gene polymorphisms have been associated with asthma, and high TLR2 expression has been shown in farming children in Europe [9, 11]. Genetic variation in TLR2 and TLR4 is a major determinant of susceptibility to asthma and allergy in rural children [12–14]. Further, the maternal TLR signaling pathway is required for protection of offspring against asthma [15]. Data from the PARSIFAL team showed that maternal exposure to microbes improves the expression of TLR genes, and affects differentiation of fetal T cells by regulating the intrauterine microbial environment, consequently playing a protective role in offspring against allergic diseases [5]. Previous studies have revealed four genetic polymorphisms in human TLR2, TLR4, and CD14 [12, 13, 16, 17], such as TLR2 rs1898830, TLR2 rs4696480, TLR4 rs4986790 and CD14 rs2569190, which play a role in the pathogenic mechanism of allergic diseases and the protection mechanism of maternal exposure to farming.

Recently, the role of Tregs in pathogenesis of asthma has been attracting increasing attention. Several studies have demonstrated decreased quantity and function of Tregs in asthma [15]. In our previous work, we showed that decreased quantity and function of Tregs in the cord blood of newborns, and it might present even prior to the Th1/Th2 imbalance in infants who had family history of allergy and were at high risk [18]. Some research has shown that common microorganisms in rural areas induce up-regulation of Tregs through the TLR pathway, subsequently showing protective role against asthma and other allergic diseases [19–21]. However, the underlying mechanisms of this protective effect remain to be determined.

Similar to the Th1/Th2 imbalance, the Treg/Th17 imbalance is also vital to the pathogenesis of asthma [22]. Moreover, the role of Th9 cells, which are characterized by IL-9 secretion and specific expression of PU.1, in development of asthma has been increasingly addressed [23, 24]; however, the association of Th9 cells with Tregs remains to be elucidated.

Since the Jilin province is a large agricultural province in China, in this study, we studied the main exposure factors in the rural environment of this area. We also attempted to investigate the effect of continual maternal exposure to the farming environment on development of the neonatal immune system, especially on the TLR2/4-Treg-Th (Th1, Th2, Th9, and Th17) axis, in the Jilin province. The results of our study will lay the theoretical foundation for allergic disease prevention in early life.

## Methods

### Study populations, maternal recruitment, and general information

One hundred and twenty-six pregnant women were recruited from the Jilin province from January 2014 to January 2016, and divided into non-farming (84 cases) and farming (42 cases) groups, with approval from the ethics committee of the Second Hospital of Jilin University. The farming group was defined as mothers living in rural areas over the last 5 years and during pregnancy, and performing routine agricultural work. The non-farming group was defined as mothers working and living away from the rural environment, having little chance of performing agricultural work. The exclusion criteria included maternal autoimmune disease history, history of medication that could affect the immune system (like systemic corticoid and immunosuppressor), infection history, and/or antibiotic usage during pregnancy, abnormal delivery process, and unhealthy newborn. Informed consent and questionnaires (items listed below) completed by the mothers before delivery were obtained.

### Analysis of endotoxin and glucans in dust from the living rooms of the pregnant mothers

Dust from the living rooms of the pregnant mothers (1 month before delivery) was collected on pre-weighed glass fiber filters, using vacuum cleaners with sampling nozzles (ALK, Horsholm, Denmark) according to a standardized protocol with photo and video instructions. The dust-containing filters were weighed and extracted in a volume of 5–40 ml, and the net dust weight determined (< 0.5 g, 5 ml; 0.5–1.0 g, 10 ml; 1.0–2.0 g, 20 ml; > 2.0 g, 40 ml). Endotoxins were measured by the kinetic chromogenic Limulus Amebocyte Lysate (LAL) test, and glucans were assayed by an inhibition enzyme immunoassay (EIA) [25].

### Monitoring the health statuses of the mothers

Maternal allergy was defined as a history of doctor’s diagnosis of asthma and/or allergic rhinitis, and/or allergic dermatitis. Total IgE measurements were performed by the RAST method using peripheral blood serum of the mothers.

### Umbilical cord blood collection and general neonatal status

Umbilical cord blood (20–30 ml) treated with anticoagulant was collected at childbirth, and used for cell isolation; the cells were cultured within 24 h of blood collection. Information on maternal gestational age and delivery type, and neonatal gender, height, body weight, and Apgar scores were obtained from medical records.

### Cytological test of umbilical cord blood

#### Isolation of cord blood mononuclear cells (CBMCs), cell culture, and analysis of lymphocyte proliferation (LP)

Cord blood samples were collected from the umbilical vein after delivery and processed within 24 h. CBMCs were isolated by density-gradient centrifugation with Ficoll-Hypaque Plus (GE Healthcare, Piscataway, NJ, USA) after dilution in phosphate-buffered saline (PBS; Gibco, Carlsbad, CA, USA). The cells were washed in RPMI 1640 (Sigma-Aldrich, St. Louis, MO, USA) and diluted in 10% human serum (Sigma-Aldrich) to a concentration of 5 × 10^6^ cells/ml. For the LP assay, 0.5 × 10^6^ cells/well were seeded and stimulated with peptidoglycan (PPG; 10 µg/ml, Sigma-Aldrich), lipopolysaccharide (LPS; 0.1 µg/ml, Sigma-Aldrich), or phytohemagglutinin (PHA; 5 µg/ml, Sigma-Aldrich) for 3 days. After incubation at 37 °C in a humidified 5% CO_2_ incubation chamber, the samples were pulsed with 1 µCi 3H-thymidine for additional 6–10 h. The cells were harvested with a ComBI cell harvester (Skatron Instrument) onto filter plates, which were read using a β-counter.

#### Measurement of CD4^+^CD25^+^FOXP3^+^T cell percentage

CBMCs were cultured for 3 days with a concentration of 1 × 10^6^ cells/ml. And then anti-human CD4–fluorescein isothiocyanate (FITC) and CD25–RPE-Cy5 (BD Biosciences) were used for surface staining, following the manufacturer’s instructions. After cell permeabilization was performed, Foxp_3_-PE/corresponding isotype control antibodies (BD Biosciences,San Jose, CA, USA) were added, and then the cells were analyzed by flow cytometry (BD FACSAria II). The gating strategy was set up as done previously [26] and is described in the Supporting Information.

#### Measurement of specific gene expression in Tregs (FOXP3, LAG-3, GITR, CTLA-4 and TGF-β) and TLR2/4

Total RNA from CBMCs after 3 days of culture was isolated using TriPure Isolation Reagent (Roch, Mannheim, Germany). Reverse transcription was performed (Invitrogen, Karlsruhe, Germany) for cDNA synthesis. Specific primer pairs were designed using the Primer Express software (Vector NTI advance10). The iCycler iQ-multicolor Real-Time PCR Detection System was used to detect the PCR product obtained by binding of SYBR Green (Applied Biosystems, Darmstadt) to dsDNA. CT indicates the number of PCR cycles required for the fluorescence signal to exceed the detection threshold value. The difference in CT values for two genes was used to calculate delta CT [ΔCT = CT (target gene) − CT (internal reference gene 18S)]. A higher ΔCT indicates lower gene expression. Here, we have presented the gene expression of FOXP3, LAG-3, GITR, CTLA-4, TGF-β, and TLR2/4 with un-stimulation and upon PHA, PPG, and LPS stimulation as ΔCT (Additional file 1: ST6). We also calculated the fold difference [fd = 2^(− ΔΔCT)]. ΔΔCT = ΔCT (a target sample-PHA/PPG/LPA stimulated) − ΔCT (a reference sample-unstimulated), and have presented the relative gene expression of the above genes (Fig. 3).

The designed primers were as follows:

- 18S rRNA: 5ʹ-AGTCCCTGCCCTTTGTACACA-3ʹ, 5ʹ-GATCCGAGGGCCTCACTAAAC-3ʹ
- Foxp3: 5ʹ-ACCTTCCCAAATCCCAGTGC-3ʹ, 5ʹ-GAAGATGGTCCGCCTGGC-3ʹ
- GITR: 5ʹ-GCACCACCCTTGTCCCCC-3ʹ, 5ʹ-CAGCGTTGTGGGTCTTGTTCC-3ʹ
- CTLA4: 5ʹ-TGGCCCTGCACTCTCCTGT-3ʹ, 5ʹ-GGACCTCAGTGGCTTTGCCT-3ʹ
- LAG-3: 5ʹ-CAATGGCGACTTTACCCTTC-3ʹ, 5ʹ-CCTCTGGGATGGGGTGTC-3ʹ
- TGF-β: 5ʹ-ACAAGTTCAAGCAGAGTACACACAGC-3ʹ, 5ʹ-CACTGCCGCACAACTCCG-3ʹ
- TLR2: 5ʹ-TTGGGGGTCATCATCAGCC-3ʹ, 5ʹ-GAGCCTGGAGGTTCACACACC-3ʹ
- TLR4: 5ʹ-ACAAAATCCCCGACAACCTCC-3ʹ, 5ʹ-AGGGCTAAACTCTGGATGGGG-3ʹ

#### Measurement of specific Th1, Th2, Th9, Th17 and Treg cytokines

The supernatant from CBMCs after 3 days of culture was collected, incubated with mixed Capture Beads (IL-9, IL-10, IL-13, IL-17, IFN-γ) (BD Cytometric Bead Array (CBA) Human Soluble Protein Master Buffer Kit),and labled with PE detection reagents. Cytokines were measured using a flow cytometer under CBA Flex Sets (BD FACSAria II).

### Analysis of the suppressive action of Tregs

CD4 + CD25- and CD4 + CD25 + T cells were isolated from the CBMCs by positive selection of CD4 + CD25 + T cells (Miltenyi Biotec, Köln, Germany). To show that the isolated CD4 + CD25 + T cells represent Tregs, we stained isolated them with either anti-human CD127-PE or FOXP3-PE, which are both important markers of Tregs. CD3- T cells were isolated (CD3-cell isolation kit, Miltenyi Biotec) and irradiated as antigen-presenting cells. The purity of the isolated CD3-, CD4 + CD25-, and CD4 + CD25 + T cells and the percentage of the CD127dim/- and FOXP3 + Tcells within the CD4 + CD25 + T cells were analyzed by flow cytometry (one sample shown in Fig. 4). The CD4 + CD25- T cells were labeled with CFSE [Cell Trace™ CFSE Cell Proliferation Kit (Invitrogen)] before culture. Then the CD4 + CD25- T cells were incubated for 3 days with irradiated CD3- cells in co-culture with/without CD4 + CD25 + T cells with PHA stimulation. Cell division of CD4 + CD25- T cells was assessed by flow cytometry by testing the CFSE content in the newly divided cells (one sample shown in Fig. 5). The cytokine concentrations in the supernatants (IFN-r, IL-13, IL-9, and IL-17) were measured using the BD Cytometric Bead Array (CBA) Human Soluble Protein Master Buffer Kit.

### Detection of gene polymorphisms of TLR2/4 and CD14

Blood Genomic DNA Midi Kit was used for DNA extraction from umbilical cord blood. Nucleotide sequences of SNPs of TLR2-15607 (rs1898830, GG, SNP) and TLR2-16934 (rs4696480, AA, SNP), TLR4 rs4986790, and CD14 rs2569190 were obtained from the NCBI gene sequence database, and Primer5.0 was used for primer design as follows:

- TLR2 rs1898830; 5ʹ-TTTAATGACTTATGAAAAAAATTACATATAAAA-3ʹ, 5ʹ-TTGTCTTGCCAGAGGTTCAT-3ʹ
- TLR2 rs4696480: 5ʹ-AGAAAAGAGAGACAATAGAACATAAAACAA-3ʹ, 5ʹ-TCACCAAGGGAGCAGTTTATT-3ʹ
- TLR4 rs4986790: 5ʹ-TTTAAATGTAATGAAAACTTGTATTCAAGG-3ʹ,
- 5ʹ-TTAACTAATTCTAAATGTTGCCATCC-3ʹ
- CD14 rs2569190: 5ʹ-CCGGCTTCCAGGCTTC-3ʹ, 5ʹ-CCAGAGAAGGCTTAGGCTCC-3ʹ

Genotyping was performed by matrix-assisted laser desorption/ionization time-of-flight mass spectrometry (Sequenom Inc., San Diego, CA, USA) [27]. PCR assays and associated extension reactions were designed using the Spectrodesigner software (Sequenom Inc.). The PCR amplification products were sequenced using the ABI3730 sequencing system (ABI). All amplification and extension reaction conditions were as previously described [28]. Deviations from the Hardy–Weinberg equilibrium (HWE) were assessed for quality control of the genotyping procedures.

### Statistical analysis

Data were analyzed using the SigmaStat1.0 software. Metric data were analyzed by either t-test [shown as means (SEM)] or Mann–Whitney rank test (shown as medians [25%/75% interquartile ranges (IQRs)]), depending on whether they were in normal distribution. One-way ANOVA and Tukey–Kramer test were performed for multiple groups. Attributes data were analyzed by either *X*^*2*^ test or Fisher’s exact test. Statistical significance was defined by P < 0.05.

## Results

### Epidemiological characteristics

Of the 126 included subjects (6 samples were withdrawn, 3 of them quit the experiment for no reason, the other 3 volunteer’s blood samples were so little to finished the following experiments), characteristics of 40 neonates with farming mothers and 80 neonates with non-farming mothers are shown in Table 1. The preliminary results showed that among farming mothers, 32 (80%) performed livestock feeding and animal husbandry as their principal occupation, 22 (55%) mainly engaged in crops related labor, 14 (35%) performed mixed farming. Farming mothers reduced almost half of their work during pregnancy, but did not change the rural living environment. There were no cases of fresh-milk drinking. There were no significant differences between the two groups regarding maternal age, height and body weight, smoking history, education, delivery type, and offspring gender, height and body weight, gestational age, and newborn Apgar scores. The contents of endotoxins in the living rooms of the farming mothers were significantly higher than those for the non-farming group (P = 0.04) (Table 1). We also conducted telephonic follow-up, which revealed that young children (aged 1.5–4.5 years) in the farming group showed a tendency of lower morbidity from allergic diseases (8.33% vs. 17.94%, shown in Additional file 1: ST1).

**Table 1:** General condition of pregnant women and newborns

	Farming group (n = 40)	Non-farming (n = 80)	P value
Mother age (year) ≠	28.82 (0.94)	30.31 (0.95)	0.28
Mother height (cm) ≠	162.32 (1.14)	160.32 (3.60)	0.64
Pre-pregnancy weight (g) ≠	66.05 (5.02)	63.07 (3.47)	0.62
Maternal smoking, n (%)			0.45
Never	29 (72.50)	64 (80.00)	
Smoking and quit before pregnancy	8 (20.00)	8 (10.00)	
Smoking before pregnancy and quit after pregnancy	3 (0)	0 (0)	
Keep smoking before after pregnancy	0 (0)	0 (0)	
Passive smoking (frequent exposure to smokers)	3 (7.50)	8 (10.00)	
Maternal allergic disease history (n %)	4 (10.00)	15 (18.75)	0.08
Allergic rhinitis (n %)	2 (5.00)	12 (15.00)	
Asthma (n %)	0 (0)	2 (2.50)	
Allergic dermatitis (n %)	2 (5.00)	3 (3.75)	
Vaginal delivery (n %)	40 (100.00)	80 (100.00)	
Maternal serum total IgE (UI/ml)#	35.5 (11.4/124.9)	51.9 (16.1/175)	0.08
Endotoxin contents in pregnant woman bedroom (EU/mg)#	44.77 (17.20/185.18)	21.73 (9.04–150.22)	*0.04*
Pregnant woman bedroom β (1,3)-d-glucan (ug/mg)#	8.78 (3.04–19.57)	5.48 (2.67–15.88)	0.10
Pregnant women close contact with livestock history, n (%)	32 (80%)	0 (0)	
Pregnant women closely contact the history of crops, n (%)	22 (55%)	0 (0)	
Pregnant women close contact with crops and livestock history, n (%)	14 (35%)	0 (0)	
Neonatal sex, boy, n (%)	21 (52.00)	40 (50.00)	0.65
Gestational age (day) ≠	257.96 (9.93)	265.81 (4.06)	0.44
Neonatal weight (g) ≠	3145.40 (120.93)	3078.06 (644.49)	0.69
Body length of newborn (cm)#	50.00 (49.25/50.00)	50.00 (48.00/50.00)	0.14
Apgar scores > 8, n (%)	40 (100)	80 (100)	

The values are represented as mean ± SEM (marked as ≠) or median (25%/75% IQR) (marked as #). T-test or Mann–Whitney rank test or X2 test were used to compare the two groups. Italic value indicates statistical significance (P < 0.05)

### Lower LPS-stimulated LP in newborns with farming mothers

**Fig. 1:**
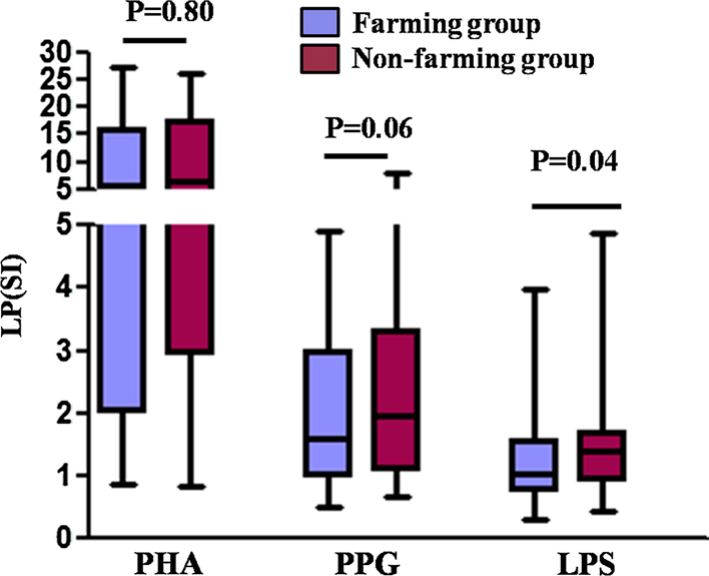
Lymphocyte proliferation was decreased by LPS stimulation in the farming group. Lymphocyte proliferation of CBMCs were assessed in the farming (n = 40) and non-farming (n = 80) groups after PHA, PPG, and LPS challenge. Proliferation was assessed by counts per minute (cpm) and then quantified by stimulation index (SI = stimulated LP (cpm)/unstimulated LP (cpm)), which was calculated as the ratio of mean cpm of stimulated over unstimulated replicates. The values are represented as median (25%/75% IQR). Mann–Whitney Rank Sum Test was used to compare the two groups. Statistical significance is indicated by P values < 0.05

There was no difference in lymphocyte proliferation (LP) in CBMCs after PHA stimulation between the two groups. However, LPS-stimulated LP in neonates with farming mother was significantly lower (P = 0.04) (Fig. 1). We did not find statistically significant difference in LP between the two groups upon PPG challenged, this could be attributed to the limited number of samples.

### Increased Th1 cell polarity as well as decreased Th2 cell polarity in newborns with farming mother, upon challenge with PPG and LPS

**Fig. 2:**
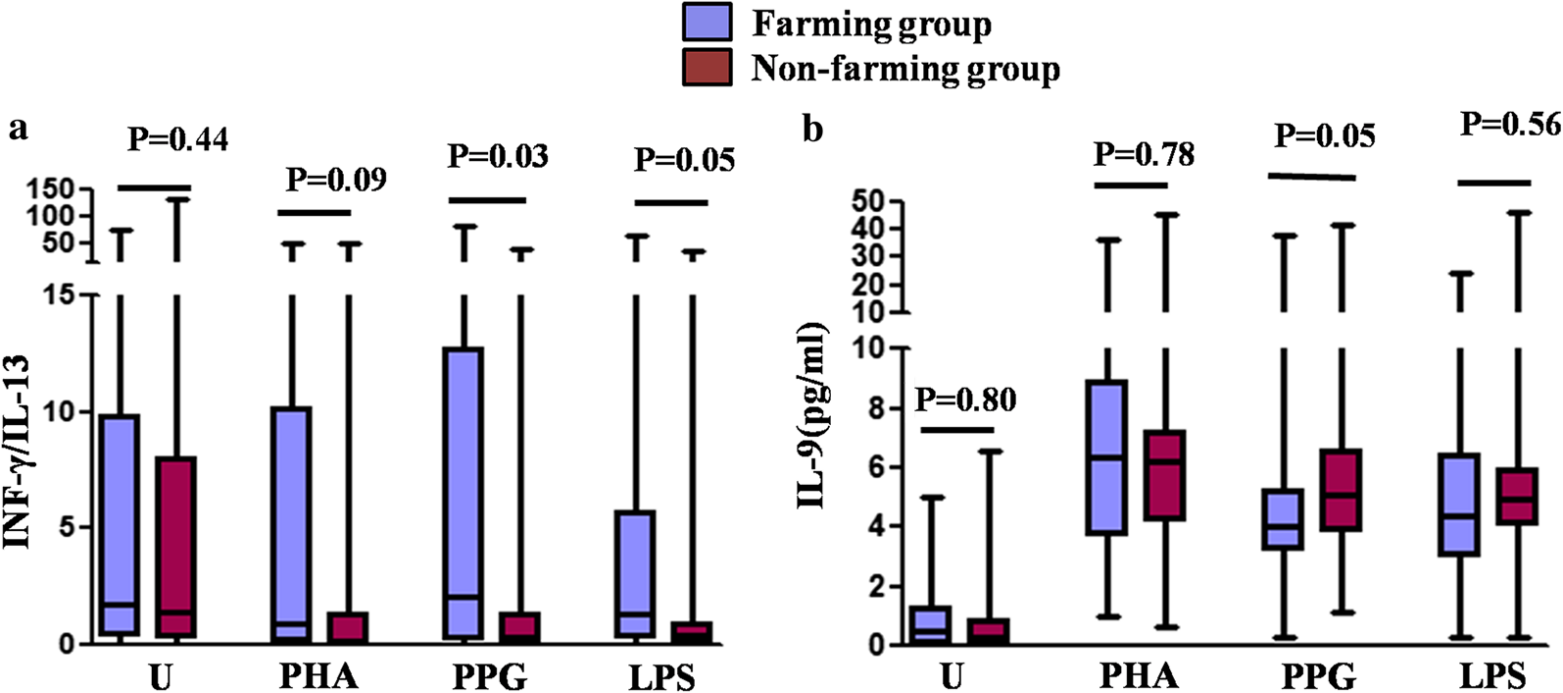
INF-γ/IL-13 was increased in the farming group after PPG and LPS stimulation, while IL-9 production was reduced after PPG stimulation. Cytokine production by CBMCs was assessed in the farming group (n = 40) and non-farming group (n = 80) after unstimulation (U), and PHA, PPG, and LPS challenge. **a** INF-γ/IL-13 ratio. **b** IL-9 production. The values are represented as median (25%/75% IQR). Mann–Whitney Rank Sum Test was used to compare the two groups. Statistical significance is indicated by P values < 0.05

IL-13 and IFN-γ are Th1- and Th2-specific cytokines, respectively. There were no significant differences in IL-10, IL-13, IFN-γ, and IL-17 secretion from neonatal CBMCs between the two groups. Upon challenge with PPG and LPS, although we did not observe statistically significant changes, neonates with farming mothers showed a tendency toward lower IL-13 production as well as higher IFN-γ production, compared with neonates in the non-farming group (Additional file 1: ST4). The IFN-γ/IL-13 ratio has been universally accepted as a marker of the Th1/Th2 ratio. Based on the previous study, we further assessed the IFN-γ/IL-13 ratio, after PPG and LPS challenge. The IFN-γ/IL-13 ratio increased significantly in the farming group (P = 0.03, P = 0.05) (Fig. 2a). IL-9 production in neonates with farming mothers was lesser than that in neonates in the non-farming group after PPG challenge (P = 0.05) (Fig. 2b).

### Up-regulation of quantity and specific gene expression of Tregs after LPS and PPG challenge in newborns with farming mothers

**Fig. 3:**
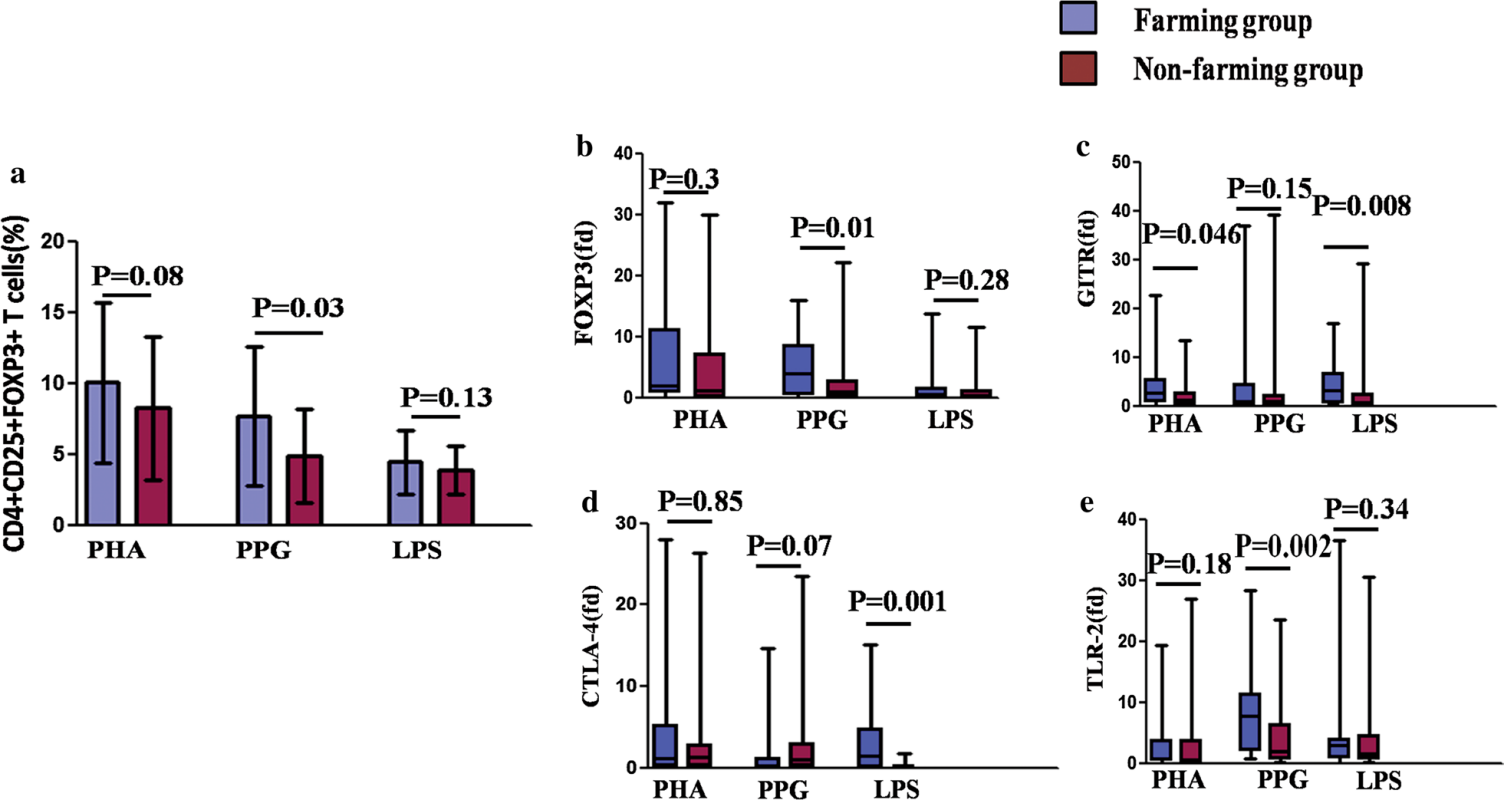
Quantity and specific gene expression of Tregs showed up-regulation in the farming group after LPS and PPG stimulation. **a** CD4 + CD25 + FOXP3 + T cell percentage in CBMCs (relative to unstimulation). The values are represented as mean ± SEM. T-test was used to compare the two groups. **b** FOXP3 gene expression in neonatal CBMCs. **c** GITR gene expression in neonatal CBMCs. **d** CTLA4 gene expression in neonatal CBMCs. **e** TLR2 gene expression in neonatal CBMCs. We used fold difference (fd) = 2^(− delta delta CT) to present the gene expression. The gating strategy used is illustrated in Additional file 1: S1. The values are represented as median (25%/75% IQR), and Mann–Whitney Rank Sum Test was used to compare the two groups. Statistical significance is indicated by P values < 0.05

Compared with that in the non-farming group, in CBMCs in the farming group, the percentage of CD4 + CD25 + FOXP3 + T cells relative to those in the unstimulated cells was higher after PPG stimulation (P = 0.03) (Fig. 3a). FOXP3, GITR, and CTLA4 are specific genes of Tregs. Compared with that in the non-farming group, in neonatal CBMCs in the farming group, FOXP3 gene expression increased after PPG stimulation (P = 0.01) (Fig. 3b); GITR gene expression also increased after PHA and LPS stimulation (P = 0.05, P = 0.008) (Fig. 3c), and CTL4 gene expression increased after LPS stimulation (P = 0.001) (Fig. 3d). Since the greatest difference had been observed after stimulation with PPG or LPS, which activated either the TLR2 or TLR4 pathway, TLR2/4 gene expression was observed in in CBMCs. Further, TLR2 gene expression increased after PPG challenge in the farming group (P = 0.002) (Fig. 3e), but TLR4 gene expression did not show any difference between the two groups.

### Suppression of Th2 cell differentiation by Tregs was enhanced in newborns with farming mothers

**Fig. 4:**
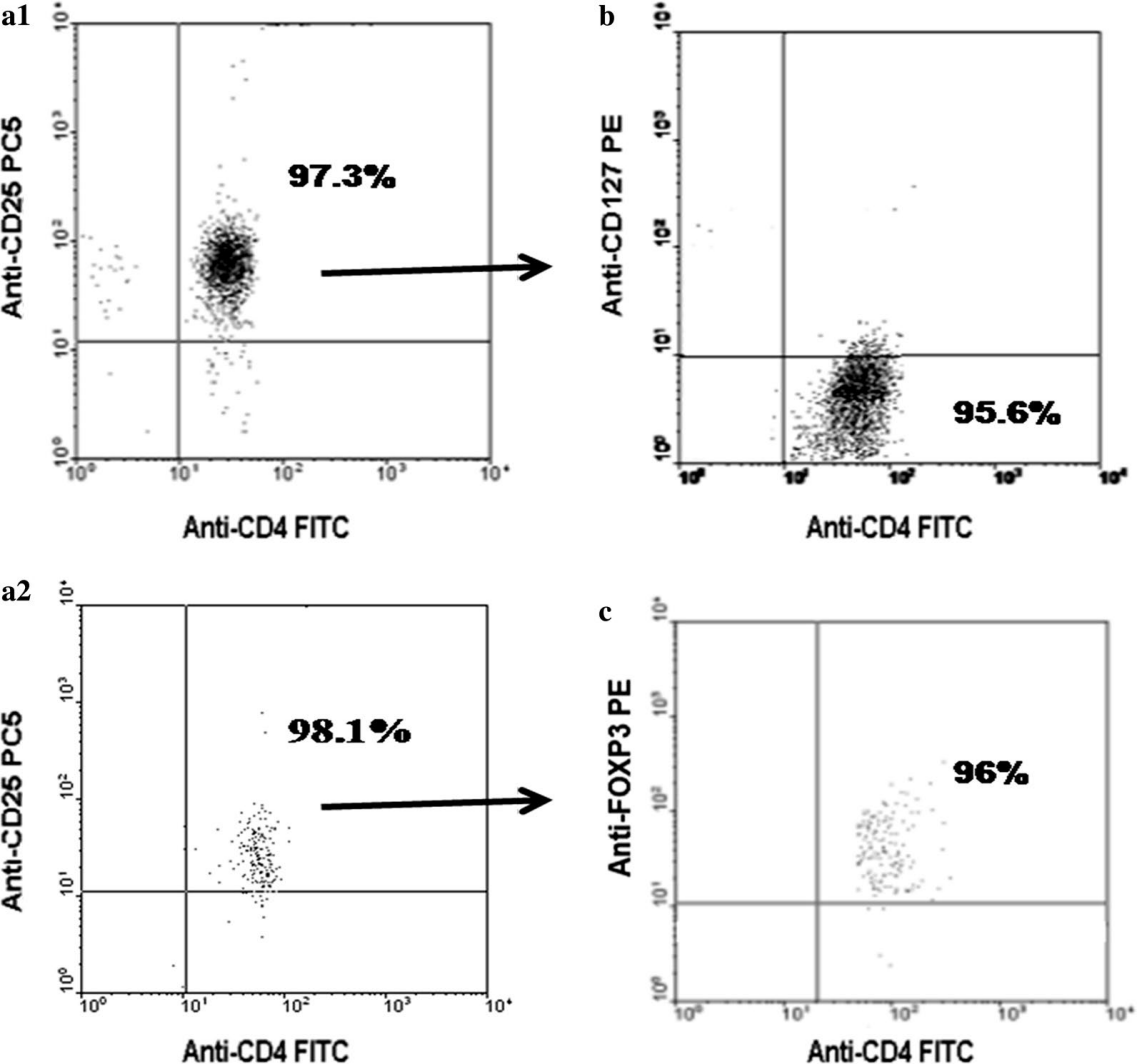
Purity of isolated CD4 + CD25 + T cells in vitro and percentage of CD127dim/- and FOXP3 + T cells in CD4 + CD25 + T cells. **a1** The purity of isolated CD4 + CD25 + T cells in one sample was 97.3%; **a2** The purity of isolated CD4 + CD25 + T cells in the other sample was 98.1%; **b** The percentage of CD127dim/- T cells in isolated CD4 + CD25 + T cells was 95.6%; **c** The percentage of FOXP3 + T cells in isolated CD4 + CD25 + T cells was 96%. n = 8. The gating strategy used is illustrated in Additional file 1: S2

**Fig. 5:**
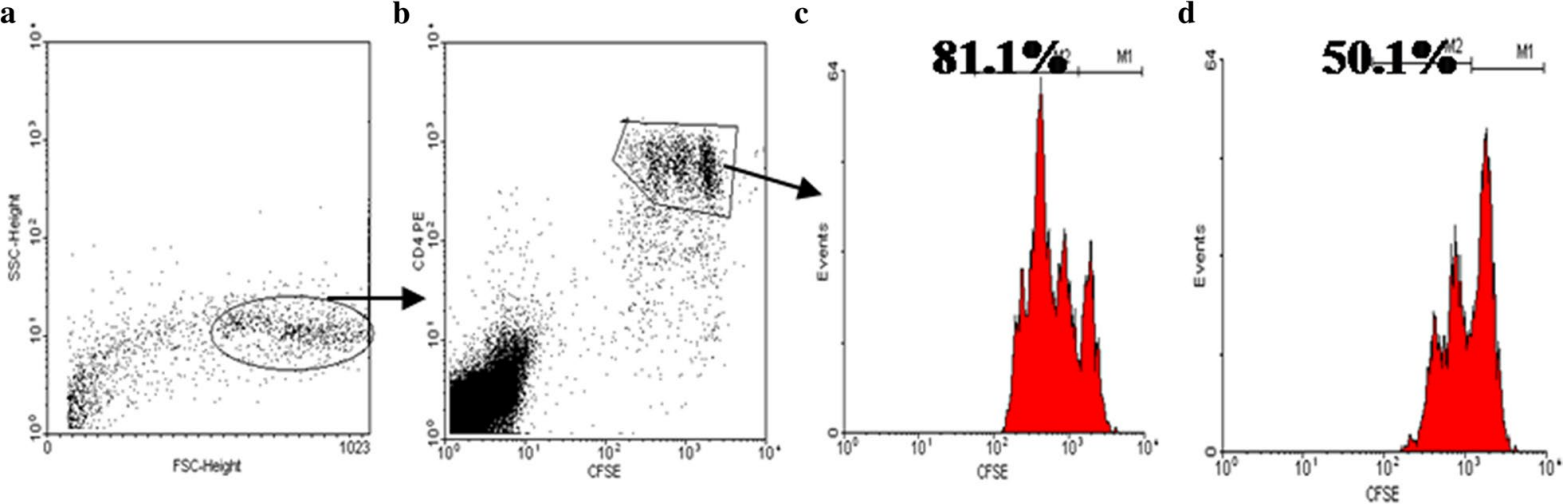
The percentage of division of CD4 + CD25- T effector cells with or without CD4 + CD25 + Tcells. CD4 + CD25- T cells were labeled using Cell Trace™ CFSE Cell Proliferation Kit before culture and cell division percentage was measured by flow cytometry after culture. **a** CBMC gate; **b** CD4 + CFSE + gate; **c** the percentage of newly divided CD4 + CD25-T cells occupied 81.1% of the total effector cells after stimulated with PHA without CD4 + CD25 + T reg cells; **d** the percentage of newly divided CD4 + CD25- T cells occupied 50.1% of the total effector cells after stimulated with PHA when cocultured with CD4 + CD25 + T reg cells. n = 8. The gating strategy used is illustrated in Additional file 1: S3

**Fig. 6:**
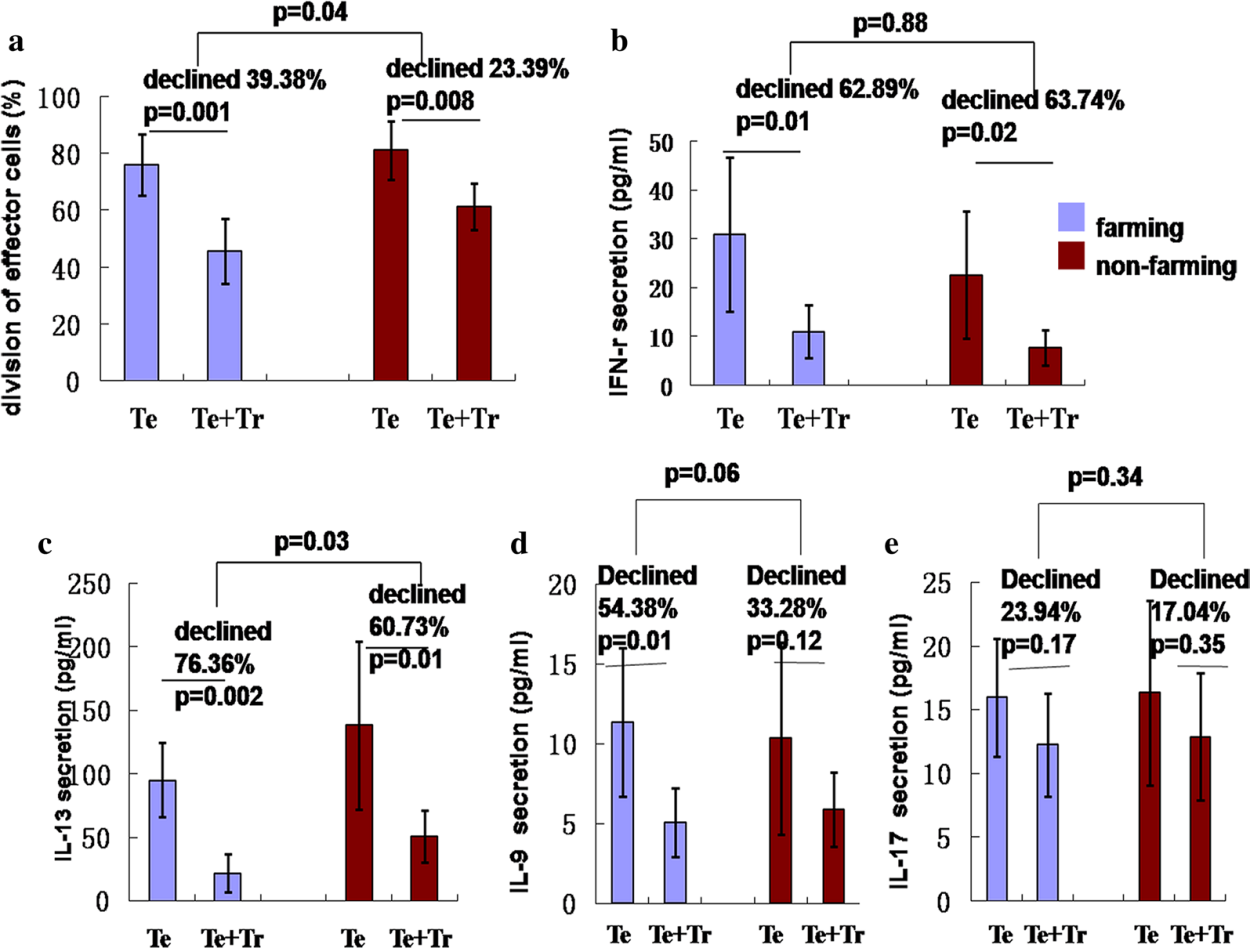
Suppressive action of CD4 + CD25 + T reg cells on the division of Th cells. **a** The suppressive action of Tregs on the division of effector T cells; **b**–**e** the suppressive action of Tregs on the production of IFN-γ, IL-13, IL-9, and IL-17 in effector T cells. Te: CD4 + CD25-T effector cell; Tr: CD4 + CD25 + T regulatory T cell (n = 8). The values are represented as mean ± SEM. T-test was used to compare the farming and non-farming groups. Statistical significance is indicated by P values < 0.05. n = 8

In isolated CD4 + CD25 + T cells, the percentage of CD127dim/-T cells and FOXP3 + T cells reached more than 95%, as revealed by flow cytometry (one sample shown in Fig. 4). The CD4 + CD25 + T cells of neonates with farming mothers showed stronger suppression of CD4 + CD25- T effector cell division (one sample shown in Fig. 5) than cells from the non-farming group (P = 0.04) (Fig. 6a). In particular, the suppression of IL-13 (Th2 cell-specific cytokine) production in CD4 + CD25- T effector cells by Tregs increased significantly in the farming group (P = 0.03) (Fig. 6c). The suppression of IL-9 (Th9 cell-specific cytokine) by Tregs tended to increase in the farming group, although not significant (P = 0.06) (Fig. 6d). However, there was no significant difference in the suppression of Th1 and Th17 cells by Tregs between the two groups.

### Increased FOXP3 gene expression and imbalance of IFN-γ and IL-13 production in farming group partly depended on the TLR2 gene polymorphisms in the newborns

**Fig. 7:**
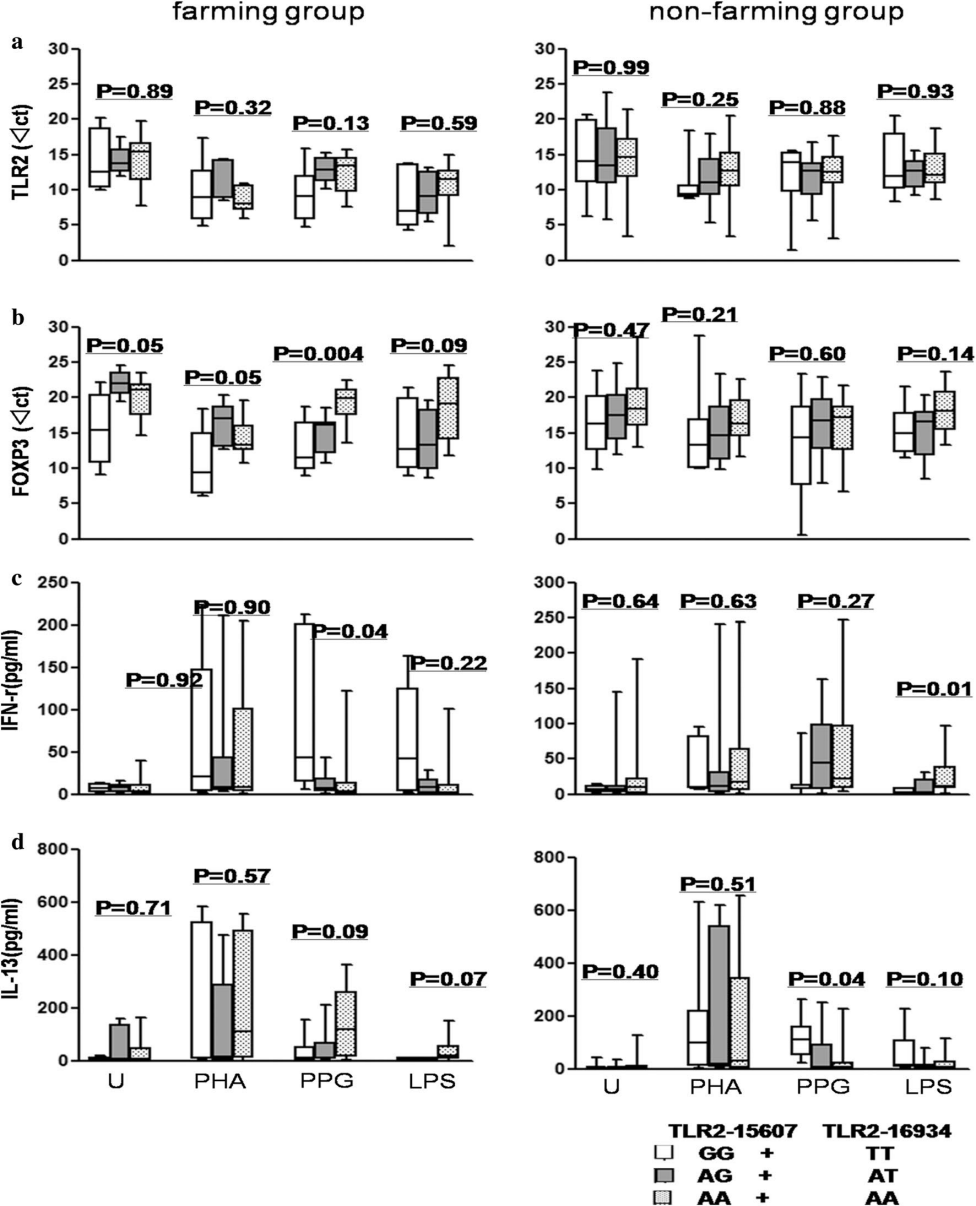
Association of TLR2 SNPs (TLR2-15607, TLR2-16934) with FOXP3 gene expression, and IFN-γ and IL-13 production. **a** TLR2 gene expression; **b** FOXP3 gene expression; **c** IFN-γ production; **d** IL-13 production. The values are represented as median (25%/75% IQR). Kruskal–Wallis test was performed to analyze three groups with alleles of GG + TT (TLR2-15607 GG + TLR2-16934 TT), AG + AT (TLR2-15607 AG + TLR2-16934 AT), and AA + AA (TLR2-15607 AA + TLR2-16934 AA). n = 40 in farming group, n = 80 in non-farming group

Gene polymorphisms of TLR2-15607 (rs1898830 GG SNP) and TLR2-16934 (rs4696480 AA SNP), but not TLR4 rs4986790 and CD14 rs2569190, were involved in the protective role of maternal exposure to farming against allergy in the offspring. The overlap rate of TT in TLR2-15607 rs1898830 and GG in TLR2-16934 rs4696480 is 100%. The overlap rate of AA in TLR2-15607 rs1898830 and AA in TLR2-16934 rs4696480 is 92.6%. The overlap rate of AT in TLR2-15607 rs1898830 and AG in TLR2-16934 rs4696480 is 94.4%. The overall rate of overlap is 97.5%. Therefore, we combined TLR2-15607 rs1898830 with TLR2-16934 rs4696480 to analyze the data. There was no significant difference in CD14 rs2569190 and TLR4 rs4986790 owing to the small number of subgroups (Additional file 1: ST2). Although the proportion of alleles in TLR2-15607 and TLR2-16934 showed no obvious difference between the two groups, depending on different allele locations, FOXP3 expression and IFN-γ and IL-13 production showed differences between the farming and non-farming groups. Unstimulated, PHA-stimulated, and PPG-stimulated FOXP3 gene expression increased (P = 0.05, P = 0.05, P = 0.004, respectively), and PPG-stimulated IFN-γ production also increased (P = 0.04) in farming newborns with G allele in the TLR2-15607 and T allele in the TLR2-16934 loci (Fig. 7). In contrast to that in the farming group, LPS-stimulated IFN-γ production decreased (P = 0.01) and PPG-stimulated IL-13 production increased (P = 0.04) in the non-farming newborns with G allele in the TLR2-15607 and T allele in the TLR2-16934 loci (Fig. 7).

## Discussion

In our studies, we found that corn cultivation, collection, and drying; and livestock, poultry, and dog feeding, but not fresh-milk ingestion were the major exposure factors in rural areas in northeast China, which was remarkable different from Loss G [4]. Endotoxin content in the living environment was significantly higher in the farming group. Farming mothers reduced their workload (by about 50%) during pregnancy, but did not change their living environment. This indicated that the increased endotoxin content (also the high microbial load) in the living environment may be the major rural exposure factor. Loss G, Carnes and Stein groups [4, 29, 30] reported that the increased endotoxin content in the environment is a protective factor against asthma and other allergic diseases. In our study, we also conducted a telephonic follow-up and found a tendency of decreased mortality due to allergic disease in young children with farming mothers. However, more information needs to be collected in future studies.

After contact with microorganisms, the immune system of newborns with farming mothers shows overall weakened responses because of the consecutive high-load microbial stimulation during pregnancy, which may be the reason why CBMCs in the farming group presented weaker proliferation responses after LPS and PPG stimulation, when compared with CBMCs in the non-farming group. We found distinctly increased IFN-γ/IL-13 ratio after LPS stimulation in neonates with farming mothers, which indicated increased Th1/Th2 ratio. Our results are consistent with the data of Siwiec [31]. Although it has been reported in previous studies that TH17 and Treg cell markers were positively correlated and influenced by maternal farm exposure [32], we did not find any notable different secretion of IL-17 and positive correlation of TH17/Tregs in the infant with farming mother. Th9 cell number has been shown to be increased in allergic diseases such as asthma [33]. We found that IL-9 secretion dropped after LPS stimulation in CBMCs from neonates in the farming group, which might be associated with their non-susceptibility to allergic diseases. Although IL-10-producing Tregs (Tr1) could secrete IL-10 [34], we did not find any change in IL-10 production in neonates with farming mothers. The reason for the above results might be that Tr1 is not the main type of Tregs in the neonatal immune system, or that IL-10 is also secreted by Th2 cells, whose down-regulation offset the enhanced IL-10 production from Tregs.

In accord with the data of Lau et al. [35], our research found that the quantity of the CD4 + CD25 + FOXP3 + Tregs and expression of FOXP3, GITR, and CTLA4 increased in newborns with farming mothers after LPS or PPG challenge. Recently, the function of neonatal Tregs had also been studied. Since in isolated CD4 + CD25 + T cells, the percentage of CD127dim/-T cells and FOXP3 + T cells reached more than 95%, isolated CD4 + CD25 + T cells were considered as Tregs in vitro to study the suppression of Thl, Th2, Th9, and Th17 cell differentiation. In accord with a previous hypothesis [18, 19], our results revealed that Tregs showed stronger suppression of Th2-specific cytokine production in the farming group, while the suppressive effect on Th1 showed no difference from that in the non-farming group. It is known that reversing the innate Th2 advantage is a difficult point in preventing allergic diseases in infants and young children, because the immune state of innate Th2 advantage date back to fetal period and lasts probably for many years, then it gradually develops to Th1 polarity so as to adapt to the surrounding environment [36, 37]. If this status, which is called “continuous fetal status of the immune system,” is not improved, the abnormality state of allergic response to foreign substances would not be eliminated [38]. The delayed maturity of the Th1 immune system is a high-risk factor for allergic diseases after birth [39]. Our study found that neonatal Th2 advantage status could also be influenced by parents and the environment. Increased exposure of pregnant women to microorganisms might reduce the risk to allergic diseases in offspring, which attributed partly to revising neonatal Th2 advantage status early by enhancing the suppressive action of Tregs on Th2 cell differentiation. Otherwise, the suppressive action of Tregs on Th9 cells tended to enhance in newborns with farming mothers (Fig. 6), which might be the reason for the declining IL-9 production. Given the important role of Th9 cells in maturation of the neonatal immune system [40], it could be a new target for early prevention and immunotherapy of allergic diseases.

Some studies [20, 41, 42] indicate that high-load of microorganisms alleviate airway inflammation, which is induced by dust mites extracts, in asthma mice. Common microorganisms in farming environment protect against asthma and other allergic diseases in animal models through modulating Tregs [19, 21]. In some clinical researches, probiotics and prebiotics have been applied during maternal pregnancy or infancy to regulate the microbiome in infants and young children, which could prevent childhood allergy [42].

TLR2/4 may bridge microbial exposure and Tregs and Th cells [35]. Based on previous studies [17], we studied four polymorphism sites on TLR2, TLR4, and CD14, and found that SNPs of TLR2-15607 and TLR2-16934 affected both neonatal Tregs and Th cells in the farming group to some extent. SNPs of TLR2-15607 and TLR2-16934 did not affect neonatal TLR2 gene expression dependent on maternal farming factor, probably because TLR2-15607 and TLR2-16934, which are locates at the TLR2 promoter regions, affected mainly TLR2 function instead of its expression [43]. The difference in the TLR2-Treg-Th axis in infants between the farming and non-farming groups might partly be due to the functional change in TLR2. More work on the detailed mechanism and follow-up on farming infants needs to be performed, aiming to delineate the influence of neonatal immune status on the susceptibility to allergic diseases during childhood.

In conclusion, endotoxin content was higher in the farming area than in the non-farming area in the Jilin province, China. Maternal living in farming environment for more than 5 years affected the innate immune system of offspring, which might partly be due to neonatal TLR2 gene polymorphism and expression. In the farming group, through the TLR2/4 pathway, maternal microbial over-load affected the quantity and function of neonatal Tregs, which regulated the early differentiation of Th1/Th2, even Th9 cells, and then reduced the susceptibility to allergic diseases in offspring. Our findings provide a new target and probable method for protecting children against asthma and other allergic diseases at the early stage. But limited to the quantities of volunteer, parts of results showed only tendency but no statistic differences. So in the future we will recruit more pregnant women as well as finish the following-up to observe the allergic status of offspring.

## Additional file

**Additional file 1.** Supporting information.

## Abbreviations

PHA: phytohemagglutinin
CTLA-4: cytotoxic T lymphocyte-associated antigen-4
PPG: peptidoglycans
LPS: lipopolysaccharide
CBMC: cord blood mononuclear cell
CBMC NF: CBMC from neonates of non-farming mothers
CBMC F: CBMC from neonates of farming mothers
Foxp3: forkhead/winged-helix family transcriptional repressor p3
GITR: glucocorticoid-induced tumor necrosis factor receptor
LAG3: lymphocyte activation gene 3
LP: lymphocyte proliferation
Tregs: regulatory T cells
TLR: toll like receptor
Th: T help
TGF-β: transforming growth factor beta
