# Supplementary material for: Maternal exposure to farming environment protects offspring against allergic diseases by modulating the neonatal TLR-Tregs-Th axis
Source: Clin Transl Allergy. 2018 Aug 17;8:34. doi: 10.1186/s13601-018-0220-0 (PMC6098605; doi:10.1186/s13601-018-0220-0)
Supplement: Supplementary file 1 — Additional file 1. Supporting information. [file 13601_2018_220_MOESM1_ESM.docx]

**Supporting Information**

Gating strategy for CD4^+^CD25^+^, CD4^+^CD25^+^Fox3^+^/CD127^-/dim^, and newly divided CD4^+^CD25^-^ CFSE^+^ cells

**S1:** To characterize CD4^+^CD25^+^ Fox3^+^ T cells, we first selected CBMCs as region1 (R1), then set R1 as gate1 (G1) to count CD4^+^CD25^+^ cells in CBMCs. Second, we selected CD4^+^CD25^+^T cells as region2 ( R2), and then set R2 as G2 to count FOXP3^+^ cells in CD4^+^CD25^+^T cells.

**S2:** The gating strategy set up as previously. To characterize CD4^+^CD25^+^ T cells, we first selected cord blood mononuclear cells (CBMCs) as region1 (R1), then set R1 as gate1 (G1) to count CD4^+^CD25^+^ cells and CD4^+^CD25^-^ cells in CBMCs. To characterize CD4^+^CD25^+^Fox3^+^/CD127^-^T cells, we selected CD4^+^CD25^+^Tcells as region2 **(**R2), and then set R2 as gate2 (G2) to count CD127^-/dim^ or FOXP3^+^ cells in CD4^+^CD25^+^T cells.

**S3:** To characterize newly divided CD4^+^CD25^-^CFSE^+^ cells, we first selected CBMCs as region1 (R1), then set R1 as gate1 (G1) to count CD4^+^CFSE^+^ cells in CBMCs, then selected CD4^+^CFSE^+^ cells as region2 (R2), and set R2 as G2 to count newly divided CD4^+^CD25^-^CFSE^+^ cells (M2) in all CD4^+^CD25^-^CFSE^+^ cells.

**ST1. The allergic status of young children followed-up**

| Allergy | Farming group  n=36 | | Non-farming  group n=78 | | | P | | |
| --- | --- | --- | --- | --- | --- | --- | --- | --- |
| all allergy | 3(8.33%) | | 14(17.94%) | | 0.09 | | |  |
| allergic wheezing | 0(0%) | | 3(3.85%) | | 0.11 | | |  |
| allergic diarrhea | 1(2.78%) | | 5(6.41%) | | 0.21 | | |  |
| allergic rhinitis | 1(2.78%) | | 6(7.69%) | | 0.15 | | |  |
| allergic dermatitis | 3(8.33%) | 7(8.94%) | | 0.45 | | |  |  |
| mix | 2(5.55%) | 6(7.69%) | | 0.33 | | |  |  |

**ST2. The frequency of gene polymorphism of TLR2-15607 (rs1898830 GG SNP), TLR2-16934 (rs4696480 AA SNP), TLR4 rs4986790 and CD14 rs2569190**

| Genes | Alleles | Farming group  n=40 | Non-farming  group n=80 | P |
| --- | --- | --- | --- | --- |
| TLR2-15607 rs1898830 | GG | 11(27.5%) | 18(22.5%) | 0.53 |
|  | AA | 10(25%) | 28(35%) |  |
|  | AG | 19(47.5%) | 34(42.5%) |  |
| TLR2-16934 rs4696480 | TT | 11(27.5%) | 18(22.5%) | 0.54 |
|  | AA | 11(27.5%) | 30(37.5%) |  |
|  | AT | 18(45%) | 32(40%) |  |
| CD14 rs2569190 | GG | 3(7.5%) | 7(8.75%) | 0.90 |
|  | AA | 5(12.5%) | 8(10%) |  |
|  | AG | 32(80%) | 65(81.25%) |  |
| TLR4 rs4986790 | AA | 40(100%) | 80(100%) |  |

**ST3 Lymphocyte proliferation of CBMC**

| [parameter](http://www.baidu.com/link?url=o_ZG5GlLZey4myoWhtna2-Sk46VG-qSnVR-vQHh_XEd7KWsOxHWbs1gpw_ftmnujCeLxVVoorzfn0b-dUIwO9sD7ZCsXLMVd1LYLQhai_Dq) | stimulus | Farming group (n=40) | Non-farming group(n=80) | P |
| --- | --- | --- | --- | --- |
| LP (SI) | PHA  PPG  LPS | 5.6(2.1/15.8)  1.58(1.03/2.98)  1.03(0.77/1.55) | 6.3(2.9/17.3)  1.95(1.11/3.27)  1.38(0.96/1.67) | 0.80  **0.06**  **0.04** |

**ST4 Cytokines production of CBMC**

|  | | Farming group(n=40) | Non-farming(n=80) | p-value | |
| --- | --- | --- | --- | --- | --- |
| IFN-γ production (pg/ml) | | | | | |
| U | | 0.83 (0.04/2.56) | 0.53 (0.12/1.85) | 0.48 | |
| PHA | | 17.05 (3.27/131.84) | 8.31 (4.82/63.59) | 0.63 | |
| PPG | | 11.55 (7.62/153.69) | 7.61 (1.73/41.52) | **0.08** | |
| LPS | | 8.43 (1.72/25.58) | 3.52 (1.24/16.88) | **0.07** | |
| IL-13 production (pg/ml) | | | | | |
| U | | 0.35 (0.07/1.49) | 0.44 (0.08/1.52) | 0.25 | |
| PHA | | 29.03 (4.54/476.54) | 58.92 (7.70/553.77) | 0.41 | |
| PPG | | 8.08 (2.61/111.30) | 16.81 (5.99/210.21) | **0.08** | |
| LPS | | 6.30 (1.86/18.00) | 8.35 (4.03/27.18) | 0.14 | |
| IL-9 production (pg/ml) | | |  |  | |
| U | 0.52 (0.09/1.27) | | 0.31 (0.01/0.88) | | 0.80 |
| PHA | 6.35 (3.73/8.73) | | 6.24 (4.23/7.17) | | 0.78 |
| PPG | 4.01 (3.30/5.09) | | 5.09 (3.90/6.51) | | **0.05** |
| LPS | 4.36 (3.18/6.41) | | 4.91 (4.16/5.92) | | 0.56 |
| IL-10 production (pg/ml) | | | | | |
| U | 1.42 (0.54/11.17) | | 1.23 (0.52/10.31) | | 0.55 |
| PHA | 147.87 (38.54/ 253.68) | | 110.66 (27.11/359.50) | | 0.94 |
| PPG | 213.59 (45.94/532.15) | | 160.86 (21.30/410.52) | | 0.14 |
| LPS | 164.49 (22.17/278.02) | | 73.48 (29.50/268.21) | | 0.18 |
| IL-17 production (pg/ml) | | | | | |
| U | | 0.12 (0.03/0.42) | 0.14 (0.03/0.73) | 0.67 | |
| PHA | | 9.26 (4.48/11.94) | 8.98 (5.15/12.30) | 0.84 | |
| PPG | | 6.59 (3.20/11.20) | 9.18 (6.20/11.20) | 0.25 | |
| PHA | | 8.42 (4.93/10.60) | 9.51 (5.56/11.11) | 0.53 | |
| IFN-γ/IL-13 | | | | | |
| U | | 1.77(0.43/9.84) | 1.45(0.35/7.97) | 0.44 | |
| PHA | | 0.89(0.14/10.11) | 0.21(0.04/1.21) | 0.09 | |
| PPG | | 2.09(0.29/12.71) | 0.37(0.06/1.29) | **0.03** | |
| LPS | | 1.32(0.37/5.05) | 0.45(0.13/0.89) | **0.05** | |

**ST5 The percentage of CD4+CD25+FOXP3+ T cells in CBMC**

|  | Farming group(n=38) | Non-farming group(n=75) | *p*-value |
| --- | --- | --- | --- |
| **CD4+CD25+FOXP3+ T cells (% in CBMCs)** | | | |
| U  PHA | 0.32 (0.08)  3.45 (1.89) | 0.41(0.07)  3.29(2.20) | 0.36  0.64 |
| PPG | 2.71(1.52) | 1.83(1.07) | **0.08** |
| LPS | 1.68 (0.61) | 1.44(0.43) | 0.30 |
| **CD4+CD25+FOXP3+ T cells (related percentage to unstimulated CBMCs)** | | | |
| PHA/U | 10.03 (5.68) | 8.23 (5.08) | **0.08** |
| PPG/U | 7.68 (4.88) | 4.86 (3.34) | **0.03** |
| LPS/U | 4.44 (2.27) | 3.87 (1.73) | 0.13 |

**ST6 Specific gene expression of Tregs and TLR2 and TLR4 in CBMC**

|  | Farming group(n=40) | Non-farming group(n=80) | *p*-value |
| --- | --- | --- | --- |
| FOXP3 gene expression (△CT to 18S) | | | |
| U | 18.00 (12.07/22.07) | 15.56 (12.83/18.19) | 0.46 |
| PHA | 13.59 (11.82/17.28) | 16.11 (10.92/20.84) | 0.36 |
| PPG | 14.40 (11.88/20.55) | 17.90 (12.07/21.56) | **0.07** |
| LPS | 18.31 (13.14/20.09) | 20.30 (17.48/22.51) | 0.11 |
| GITR gene expression (△CT to 18S) | | | |
| U | 12.78 (5.39/ 14.75) | 11.80 (6.41/15.18) | 0.96 |
| PHA | 9.63 (7.04/ 12.68) | 12.43 (9.38/17.33) | 0.14 |
| PPG | 12.82 (10.17/ 15.41) | 12.83 (8.11/14.67) | 0.47 |
| LPS | 9.10 (3.72/ 12.92) | 12.06 (9.52/15.20) | **0.06** |
| CTLA4 gene expression (△CT to 18S) | | | |
| U | 19.81 (16.01/ 23.15) | 17.80 (6.95/19.75) | 0.44 |
| PHA | 19.87 (15.21/21.66) | 13.65 (11.88/19.06) | 0.17 |
| PPG | 20.63 (18.65/26.49) | 15.55 (11.61/21.98) | 0.10 |
| LPS | 16.69 (11.13/18.25) | 21.09 (18.65/23.01) | **0.03** |
| LAG-3 gene expression (△CT to 18S) | | | |
| U | 17.17 (6.37/22.30) | 15.31 (10.80/19.68) | 0.66 |
| PHA | 12.27 (4.34/15.87) | 15.84 (9.03/20.76) | 0.09 |
| PPG | 16.48 (14.84/19.98) | 13.36 (9.31/21.10) | 0.32 |
| LPS | 15.92 (13.01/19.06) | 19.15 (6.54/22.11) | 0.48 |
| TGF-b gene expression (△CT to 18S) | | | |
| U | 16.00 (12.05/ 22.31) | 15.46 (11.25/19.23) | 0.89 |
| PHA | 14.16 (11.64/18.73) | 18.42 (15.28/21.04) | 0.15 |
| PPG | 13.56 (10.83/21.36) | 15.54 (12.40/18.07) | 0.87 |
| LPS | 17.96 (10.35/19.45) | 18.22 (15.66/21.53) | 0.71 |
| TLR2 gene expression (△CT to 18S) | | | |
| U | 11.35 (10.65/12.9) | 11.5 (10.21/12.09) | 0.76 |
| PHA | 12.4 (11.35/14.87) | 13.25 (12.54/14.65) | 0.28 |
| PPG | 8.85 (7.21/11.88) | 11.3 (8.74/12.76) | **0.04** |
| LPS | 9.75 (7.51/12.37) | 11.35 (10.3/11.82) | **0.12** |
| TLR4 gene expression (△CT to 18S) | | | |
| U | 12.85 (11.51/13.76) | 11.97 (9.72/12.11) | 0.54 |
| PHA | 12.6 (11.87/12.73) | 12.07 (11.01/13.41) | 0.66 |
| PPG | 12.3 (11.71/13.0) | 11.79 (10.81/13.32) | 0.41 |
| LPS | 11.75 (11.06/13.15) | 9.04 (8.46/12.0) | 0.10 |
